# Prediction of Extensibility and Toughness of Wheat-Flour Dough Using Bubble Inflation–Structured Light Scanning 3D Imaging Technology and the Enhanced 3D Vgg11 Model

**DOI:** 10.3390/foods14081295

**Published:** 2025-04-08

**Authors:** Xiuzhi Luo, Changhe Niu, Zhaoshuai Zhu, Yuxin Hou, Hong Jiang, Xiuying Tang

**Affiliations:** 1 College of Mechanical Engineering, Xinjiang University, Urumqi 830046, China; lxz_shz@163.com; 2 Institute of Agricultural Mechanization, Xinjiang Academy of Agricultural Sciences, No. 291, Nanchang South Road, Shayibak District, Urumqi 830046, China; xjnch@xaas.ac.cn (C.N.); zhuzhaoshuaixj@163.com (Z.Z.); 3 College of Engineering, China Agricultural University, No. 17 Qinghua East Road, Beijing 100083, China; houyuxin980929@163.com

**Keywords:** 3D CNN, dough extensibility and toughness, bubble inflation, 3D scanning imaging technology, CBAM, point cloud images

## Abstract

The extensibility of dough and its resistance to extension (toughness) are important indicators, since they are directly linked to dough quality. Therefore, this paper used an independently developed device to blow sheeted dough, and then a three-dimensional (3D) camera was used to continuously collect point cloud images of sheeted dough forming bubbles. After data collection, the rotation algorithm, region of interest (ROI) extraction algorithm, and statistical filtering algorithm were used to process the original point cloud images. Lastly, the oriented bounding box (OBB) algorithm was proposed to calculate the deformation height of each data point. And the point cloud image with the largest deformation depth was selected as the data to input into the 3D convolutional neural network (CNN) models. The Convolutional Block Attention Module (CBAM) was introduced into the 3D Visual Geometry Group 11 (Vgg11) model to build the enhanced Vgg11. And we compared it with the other classical 3D CNN models (MobileNet, ResNet18, and Vgg11) by inputting the voxel-point-based data and the voxel-based data separately into these models. The results showed that the enhanced 3D Vgg11 model using voxel-point-based data was superior to the other models. For prediction of dough extensibility and toughness, the *R_p_* was 0.893 and 0.878, respectively.

## 1. Introduction

As is well known, wheat flour is the most fundamental and important raw food material for all of humanity. Dough is made from flour and is an intermediate product of all flour products. Furthermore, the rheological properties of dough largely determine the quality of the final dough products. The toughness and extensibility of dough are important indexes to measure the rheological properties of dough [1]. The requirements for the extensibility and toughness of the dough are different when making different types of flour-based foods [2,3]. Therefore, the bakery industry, bread and pastry factories, and some researchers attach great importance to the detection of dough extensibility and toughness.

The most common detection methods for dough extensibility and toughness are from the perspective of mechanical stretching [4,5,6,7,8,9]. In addition, since the 1920s, devices such as the Alveograph^®^ and the Alveolab^®^ (Chopin Technologies, Paris, France) or the inflation rig fixed to the TA.XTPlus texture analyzer (Stable Micro Systems Ltd., Godalming, UK) based on the bubble inflation technique [10] have been used to characterize wheat flours at the ambient temperatures used in the bakery industry and to evaluate the quality of final dough products. However, this technique did not link the spatial deformation of the dough bubble with its rheological properties. Scholars [1,10,11] have confirmed that the deformation of dough bubbles is indeed closely related to their rheological properties.

In recent years, the laser airflow detection technique has been successfully applied in the quality testing fields of kiwifruit, meat, and bread [12,13,14]. In addition, Asaithambi et al. [3] used an air-jet impulse system to detect the rheological properties of bread dough for the sake of industrial dough quality control. The testing principle of this technique is to apply airflow pressure to the surface of food to cause deformation, and then laser distance sensors measure the depth of the deformation. However, this technique cannot obtain the spatial deformation under the action of airflow pressure [15]. The combination of pulse air puffs and structural light in 3D imaging can address the drawbacks of this technology. Lu et al. [16] first proposed a pulse air puff combined with structural light 3D imaging to detect the tenderness of beef. Based on this paper, Luo et al. [15] developed a complete detection system based on the 3D airflow scanning imaging technique to detect the texture of beef. Dedey et al. [10] combined bubble inflation with a magnification digital microscope to observe the biextension of dough and gluten films at the top of a bubble. These studies further demonstrated the potential of combining airflow and machine vision to examine the mechanics underlying the mechanical rupture of dough films. Based on this theoretical knowledge, Luo et al. [1] used 3D airflow imaging technology combined with sheeted dough blowing to detect the extensibility and toughness of the dough. However, point cloud data collected using this technology required manually crafting features by using the spatially or geometrically calculated statistics of point clouds. Furthermore, it is challenging to generalize them to other areas [17]. Convolutional neural network (CNN) models with strong representation power and generalization capability can effectively avoid hand-crafted feature extraction [18].

The CNN model is a type of artificial neural network that aims to handle and analyze large amounts of data by creating a feature-learning network that resembles the human brain. It can automatically extract linear and nonlinear features from raw data and achieve end-to-end modeling advantages [19]. In addition, the ability of CNNs to automatically extract features provides technical support for image information encoding. Thus, CNNs have been successfully applied in the field of food testing using images [20]. Inspired by the application effect of CNNs in image feature extraction, some studies employed CNNs [21,22] to process the projected images after projecting 3D point clouds onto 2D images. However, the 3D spatial structural information of point clouds will be lost with this direct projection method. Furthermore, accurately classifying large-scale point clouds is a difficult challenge because of their chaos, sparsity, and irregularity. Compared with point-based methods, the voxelization method is applied to the point cloud, and voxel-based 3D CNN models for prediction [23,24] have shown promising results. In order to maintain the advantages of voxel-based methods and reduce the complexity and burden of the computation of voxel-based 3D CNNs, some scholars combined points and voxels to solve these problems. These studies paralleled [25,26] point- and voxel-based methods to better exploit the advantages of point- and voxel-based methods, but this method brings a substantial computational burden and fails to learn real key points [26]. Song et al. [27] converted unstructured point cloud data to structured data by voxelization. Then, the voxel-based data were sampled using a random sampling method and put into a 3D CNN to achieve good detection results.

The attention mechanism is proposed via an analogy to the human perceptual process, in which a region of interest is often attended to instead of the entire scene. In different attention mechanisms, the channel attention mechanism can adaptively adjust the weights of different channels of the input tensor, making the model pay more attention to some important channels. For example, the SENet [28] attention algorithm includes a global average pooling layer and two fully connected layers. However, when there are too many parameters, the training efficiency is greatly reduced. The spatial attention mechanism can adaptively adjust the weights of different positions in the input tensor, making the model pay more attention to some important positions. However, because only the weight of the spatial position can be adjusted and the weights of different channels cannot be adjusted, the effect is poor when processing channel features [29]. In addition, unlike in the 2D image task, the input is a structured image. For point cloud data, the input data are usually unordered. Therefore, this study used a three-dimensional CBAM, which can simultaneously adjust weights in both the spatial and channel dimensions to improve the performance of the model. At present, some scholars [28,30] have successfully applied the three-dimensional attention mechanism. Thus, in this study, the point cloud data were voxelated as points to input into the Vgg11 3D CNN. In addition, these studies [30,31] have reported that introducing the CBAM [32] into the model can effectively improve the predictive accuracy of the model. Therefore, the CBAM was embedded in Vgg11 to further improve its predictive performance.

The specific objectives of this study were as follows: (1) obtaining continuously collected point cloud data using self-developed detection system devices; (2) finding the frame image with the maximum deformation depth in the continuously collected bubble point cloud images as the data for this study; (3) integrating the CBAM into the Vgg11 model to improve the model performance; and (4) putting voxel-point-based data and voxel-based data into the four different 3D CNN models, respectively, and comparing their performances for predicting the extensibility and toughness of wheat-flour dough.

## 2. Materials and Methods

### 2.1. Self-Developed Data Acquisition System

**Figure 1:**
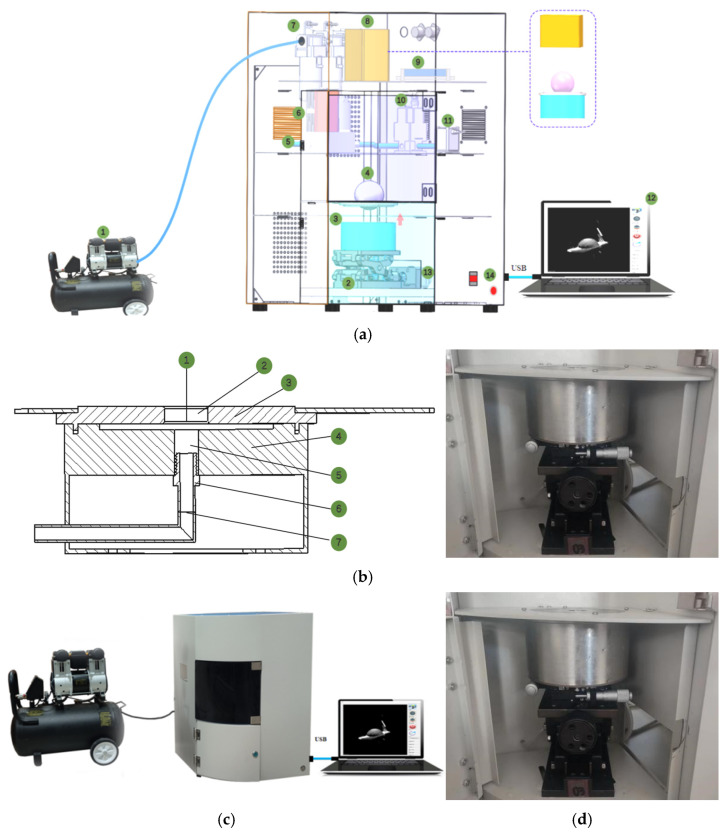
(**a**) Schematic of airflow structured light 3D imaging technology system: 1. an air compressor; 2. an automatic lifting platform; 3. a self-developed bubble blower; 4. a bubble blown by sheeted dough; 5. an air tube; 6. a flowmeter; 7. a dual air filter; 8. a 3D camera; 9. a voltage and current analogue input and output module; 10. an electro-pneumatic proportional valve; 11. a solenoid valve; 12. a computer; 13. electrical machinery; and 14. a power switch. (**b**) Self-developed bubble blower: 1. small round patch of dough; 2. blowing mouth; 3. upper plate; 4. lower plate; 5. air outlet; 6. joint; and 7. trachea. (**c**) Collected physical images of the system. (**d**) Developed software acquisition system.

The independently developed acquisition system (Figure 1a) mainly includes the following parts [1]: 1. an air compressor (2-pole 2200, OTUs Corporation, Taizhou, China); 2. an automatic lifting platform; 3. a self-developed bubble blower; 4. a bubble blown by sheeted dough; 5. an air tube; 6. a flowmeter (ACU10FA-LC, Beijing, China); 7. a dual air filter; 8. a 3D camera (M05G016-200, Shenzhen, China); 9. a voltage and current analogue input and output module (JYDAMAIAO, Beijing Central Control Intelligent Technology Corporation, China); 10. an electro-pneumatic proportional valve (ITV2030-21F2L; SMC Corporation, Tokyo, Japan); 11. a solenoid valve (VX220LA; SMC Corporation, Tokyo, Japan); 12. a computer (Intel (R) Core (TM) i5-7300HQ); 13. electrical machinery (JGB37-520, DC12V45RPM, Shenzhen, China); and 14. a power switch. Furthermore, Figure 1b is the self-developed bubble blower and mainly consists of the following parts: 1. small round patch of dough; 2. blowing mouth; 3. upper plate; 4. lower plate; 5. air outlet; 6. joint; and 7. trachea. The collected physical images of the system are shown in Figure 1c.

This collection and control system was developed using Visual Studio 2017 (VS 2017) software with the PCL1.8 configuration on the Windows 10 operating system, as shown in Figure 1d. The human–computer interaction interface was written and developed using the C++ programming language in the QT plugin of version. 2017. The development of this software system included two parts: an airflow control system and a 3D point cloud acquisition system. The airflow control system was controlled by the AIAO module, which outputs the control voltage through the Modbus RTU protocol.

### 2.2. Sample Preparation

This study selected high-gluten, medium-gluten, and low-gluten wheat flour (Luzhong Ktie Flour Co., Ltd., Weifang, China) as the research materials. The biggest difference between high-gluten, medium-gluten, and low-gluten wheat flour is their varying gluten protein content. The protein contents of high-gluten, medium-gluten, and low-gluten wheat flour were 12.2%, 11.5%, and 8.5%, respectively. In order to form dough with different rheological properties, this study added gluten and starch to these three types of flours, respectively. The types of gluten and starch, their ratio to flour, and the preparation process of sheeted dough were made based on a previous paper published by our team [1]. A total of 87 doughs with different textures and rheological properties were prepared, with six slices prepared for each dough. Three of the sheeted dough samples were used for bubble inflation testing by the development of the device. The other three sheeted doughs were used to measure their extensibility and resistance towards extension (toughness) by the XT Plus texture tester (TA. XT Plus, Stable Micro System, London, UK).

### 2.3. Data Collection

**Figure 2:**
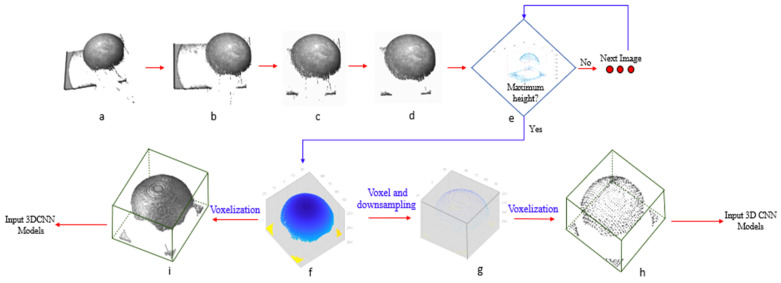
Schematic diagram of the preprocessing process of point cloud images. (**a**) Original point cloud image; (**b**) image rotated around the *y*-axis; (**c**) region of interest; (**d**) point cloud image filtering and denoising; (**e**) point cloud image with the highest deformation height; (**f**) the selected point cloud image; (**g**) the data were processed via voxel downsampling; (**h**) the voxel-point-based data was voxelated to 224 × 96 × 96. (**i**) The data were voxelated to 224 × 96 × 96.

Before the sheeted dough was placed on the bottom plate of the self-developed bubble blower, vegetable oil was applied to the bottom plate [1]. And then the lifting platform was adjusted so that the upper plate and the lower plate were aligned and tightened (Figure 1c). The measurement accuracy of the structured light scanning camera was 0.02 mm, the frame rate was 0.6 s, and the field of view was 40 × 70 mm. The flowmeter’s operating time and rate in this study were 15 s and 0.6 L/min, respectively. The airflow caused the sheeted dough to expand and form a bubble. At the same time, the 3D camera continuously scanned and collected the point cloud data of the dough bubbles, and the images are shown in Figure 2a of this paper [1]. This study involved 87 dough samples, each of which underwent three or four bubble inflation tests.

The other three sheets of dough were used to measure its standard value using the XT Plus texture tester. The pastry extensibility mode was selected as the measurement mode, and a p/0.5 S spherical probe was used as the test probe. The test speed was set to 0.5 mm/s, the pretest speed was set to 2 mm/s, the post-test speed was set to 2 mm/s, and the trigger force was set to 5 g. Before conducting the data collection, the sheeted doughs were wrapped in cling film and placed in the thermostatic chamber at about 25 °C for 20 min [1].

### 2.4. Point Cloud Image Preprocessing

The preprocessing process of point cloud images is shown in Figure 2. Firstly, the original point cloud image (Figure 2a) needed to be rotated 63 degrees in the opposite direction of the *y*-axis to make it parallel to the x0y plane (Figure 2b). The second step was to extract the region of interest of the dough bubble (Figure 2c) [1]. After this step, the statistical filtering algorithm (Statistical Outlier Removal) was proposed to remove the noise from the extracted region-of-interest point cloud data. In this study, the optimal Num-Neighbors and threshold were set to 100 and 0.8, respectively (Figure 2d). Fourthly, the oriented bounding box (OBB) algorithm [16] was used to obtain the deformation height of each preprocessed point cloud image (Figure 2e). And the data with the highest deformation height were used as the required point cloud image for this paper (Figure 2f).

Before inputting data into the different 3D CNN models, the processed data were needed to build an occupancy grid from a point cloud and spatially bin it into a 124 × 96 × 96 voxel grid. The binary occupancy grid strategy was adopted, with 1 indicating the voxel that was inside the surface and 0 indicating a voxel outside the surface [27]. In order to significantly reduce the computation burden and detection bias and improve the prediction accuracy, this paper voxelated the obtained preprocessed point cloud and then downsampled it (Figure 2g). To verify whether voxels as point data affected the predictive performance of the models, this study inputted voxel-based data (Figure 2i) and voxel-point-based data (Figure 2h) into the same models and compared the training and testing performances of the two data-processing methods.

### 2.5. The 3D CNN Model Design

#### 2.5.1. The 3D Vgg11 Model

The Vgg network [33] is an improvement in the AlexNet network structure and has been experimentally proven to be superior on the ImageNet dataset. The Vgg11 network structure is a network model structure proposed based on the Vgg16 network by changing the depth and width of the model, and it consists of 8 convolutional layers, 5 max pooling layers, and 3 fully connected layers. This network has both shallow depth and strong expressive and feature extraction capabilities [29]. In addition, 2D Vgg11 differs from 3D Vgg11 in that 3D convolution has an additional depth dimension. Therefore, the convolutional kernel needs to perform sliding window operations on the spatial dimensions (length, depth, and width) of the input 3D image. *L*, *D*, and *W* represent the length, depth, and width of voxel data. Each time the sliding window is related to the values in the (*k_l_*, *k_d_*, *k_w_*) window, a value in the output 3D image is obtained as the final output 3D feature map.

(1) Convolutional layer

The stride size of each convolution is *s_l_* × *s_h_* × *s_w_*. The convolution output is given as follows:

(1) $$L_{out}=\frac{L_{in}+2p-k_{l}}{s_{l}}$$

(2) $$W_{out}=\frac{W_{in}+2p-k_{w}}{s_{w}}$$

(3) $$D_{out}=\frac{D_{in}+2p-k_{d}}{s_{d}}$$

where *L_in_*, *W_in_*, and *D_in_*, respectively, represent the length, width, and depth of the input 3D Vgg model data; *P* represents filling; *s_l_*, *s_w_*, and *s_d_*, respectively, represent the step size of each 3D convolution; and *k_l_*, *k_w_*, and *k_d_* are the convolutional kernels of 3D convolutional neural networks; the stride and 3D convolution kernel for each convolution were set to 1 × 1 × 1 and 3 × 3 × 3, respectively.

(2) Activation function

The output of the convolution layer is passed to an activation function, which is to determine whether and how much information is transmitted. In this study, *ReLU* = *max* (*x*, *0*) was selected as an activation function, and its expression is as follows:

(4) $$R(x)=\left\{\begin{array}{c} x\\ 0 \end{array}\right.\begin{array}{cc} & x<0\\ & x\leq 0 \end{array}$$

(3) Pooling layer

The max pooling of 3D, which has a kernel size of *K_l_* × *K_h_* × *K_w_*, and a stride size of *s_l_* × *s_h_* × *s_w_* are used. The following is the equation to calculate the max pooling output:

(5) $$M(u,v,w)=\max_{c=0\ldots x-1,\;\;d=1,\ldots ,y-1,e=0\ldots z-1}O(u+c,v+d,w+e)$$

The output size of the max pooling matrix is *M* (*u*, *v*, w), with *f* length, *g* height, and *h* width, in which *f* is equal to (*L − K_l_*)/*s_l_* +1, *v* is equal to (*H* − *K_h_*)/*s_h_* + 1, and *w* is equal to (*W* − *K_w_*)/*s_w_* +1, where *u* runs from 1 to *f*, *v* runs from 1 to *g*, and *w* runs from 1 to *h*. The kernel size and stride size are *2* and 1, respectively.

(4) Fully connected layer

The output of the max pooling layer is combined together to form a feature input vector with a size of 512 × 1 in the fully connected layer to train backpropagation with dough toughness and extensibility prediction. This study uses serialization operations to merge the three connection layers together and package them into a new module. To prevent overfitting of the model and improve its generalization ability, this model added a dropout layer to the fully connected layer, with a random deactivation ratio set to 0.5. In addition, the BN layer [34] was embedded in the 3D Vgg11 model to improve the performance of the model.

#### 2.5.2. Enhanced 3D Vgg11 Model

**Figure 3:**
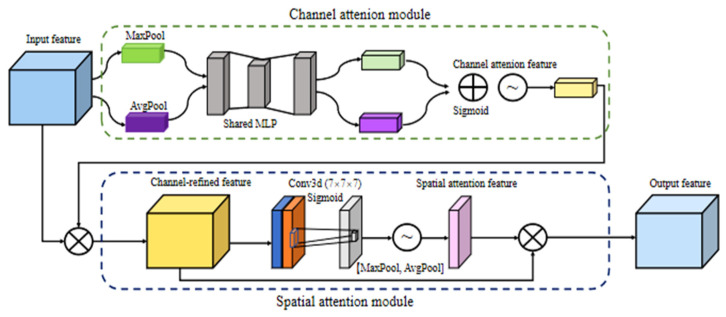
Schematic diagram of 3D Convolutional Block Attention Module (CBAM) attention mechanism structure. Note: MaxPool and AvgPool are the max pooling and average pooling with a kernel size of 1 × 1 × 1; “⊕” represents adding the values of two channels; “
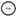
” is feature mapping; “⊗” represents the multiplication of the values of two channels.

The enhanced 3D Vgg11 proposed in this paper added a 3D version of the Convolutional Block Attention Module (CBAM) to its network structure [32]. The 3D Convolutional Block Attention Module (CBAM) was chosen for processing in this study due to the presence of some irrelevant external noise and other influencing factors in the recognition image. It is composed of two parts: the channel attention module and the spatial attention module. The channel attention module was mainly used to weigh the attention of the input channel dimension to improve the model’s attention to different channels. The schematic diagram of 3D CBAM is shown in Figure 3.

The final output, after passing through the attention mechanism, is:

(6) $$y_{a}^{\prime \prime }=s\cdot y_{a}^{\prime }$$

(7) s=σ(a)

(8) $$m=f(Avgpool(y_{a}^{\prime })+Maxpool(y_{a}^{\prime }))$$

where *m* is a three-dimensional feature map, *f* is a convolutional layer, and *s* is a spatial attention weight; *σ* is a sigmoid activation function, and *y_a_* is the channel attention output.

(9) $$y_{a}^{\prime }=s_{a}\cdot y_{a}$$

(10) $$s_{a}=\sigma (f(MLP(Avgpool(y_{a}^{\prime }))+MLP(Maxpool(y_{a}^{\prime })))$$

(11) $${\hat{y}}_{aijk}=\frac{y_{aijk}-\mu_{a}}{\sqrt{\sigma_{a}^{2}+\varepsilon }}$$

Here, a is the channel index, and *i*, *j*, and *n* are the spatial indexes; ε is a very small constant; MLP is a multi-layer perceptron; AvgPool and MaxPool represent average pooling and maximum pooling, respectively; σ is a sigmoid function; *S_a_* is the channel attention weight; $\mu_{a}$ and $\sigma_{a}^{2}$ are the mean and variance, respectively. Its specific expression is:

(12) $$\mu_{a}=\frac{1}{D\times W\times L}\sum_{i=1}^{D}\sum_{j=1}^{W}\sum_{k=1}^{L}Y_{i,j,n}$$

(13) $$\sigma_{a}^{2}=\frac{1}{D\times W\times L}\sum_{i=1}^{D}\sum_{j=1}^{W}\sum_{k=1}^{L}\left(y_{ijn}-\mu_{a}^{2}\right)$$

where *H*, *W*, *L*, and *C* represent the height, width, length, and number of channels of the feature map.

### 2.6. Network Training

**Figure 4:**
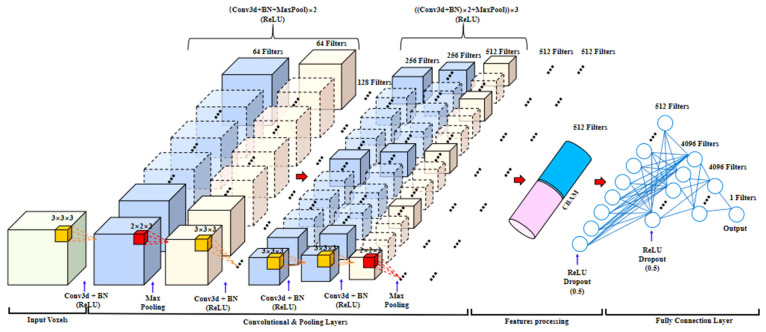
Structure diagram of enhanced Vgg11 model.

The learning rate in this study was adjusted using Adam optimization. The stochastic gradient descent algorithm was extended by the Adam optimization algorithm. It has been extensively employed in deep learning applications lately, especially in natural language processing and computer vision. Through first-order data estimation and second-order gradient moment estimation, the Adam method adaptively modifies the learning rate of each parameter [35]:

(14) $$m_{t}=\beta_{1}m_{t-1}+(1-\beta_{1})g_{t}$$

(15) $$v_{t}=\beta_{1}v_{t-1}+(1-\beta_{1})g_{t}^{2}$$

(16) $$M_{t}=\frac{m_{t}}{1-\beta_{1}^{t}}$$

(17) $$V=\frac{v_{t}}{1-\beta_{2}^{t}}$$

(18) $$\vartheta_{t+1}=\vartheta_{t}-\frac{\sigma }{\sqrt{V_{t}+\varepsilon }}M_{t}$$

where *m_t_* and *m_t_*_−1_ are the first-moment estimates of the gradient at time *t* and *t*−1, respectively; *v_t_* and *v_t_*_−1_ represent the second-moment estimates of the gradient at time *t* and *t*−1, respectively; *g_t_* is the parameter gradient *ϑ* at time *t*; and *M_t_* and *V_t_* are the corrections of *m_t_* and *v_t_*, respectively. The BN layer and 3D CBAM were embedded in the 3D Vgg11 model to improve the performance of the model, and its network structure is as follows (Figure 4):

The 3D CNN models were implemented using Python 3.10 with the Pytorch deep learning library. And all our experiments were carried out on a personal computer (Inter (R) Core (TM) i7-7700HQ CPU@2.80GHz) with a single NVIDIA GeForce GTX 1050. In the proposed different 3D CNN models of this study, the learning rate was adjusted using Adam optimization, and the weight decay and drop rate are set to 0.001 and 0.5, respectively. Furthermore, the initial learning rate was 0.001, and the epoch count was 50, respectively.

### 2.7. Performance Evaluation of the Models

The correlation coefficient (R) of calibration (R_c_), validation (R_v_), and prediction (R_p_), as well as the root mean square error (RMSE) of calibration (RMSEC), validation (RMSEV), and prediction (RMSEP), were used to assess the impact of regression modeling. The mean squared error (MSE) was an indicator used to evaluate the changes in loss values during the model training process. Furthermore, the residual predictive deviation (RPD) and mean absolute error (MAE) were also important indicators to evaluate the prediction set. Generally, the reliability of the regression model’s results is indicated by a higher *R* value and a lower RMSE value. According to Bonin et al. [36], an RPD value of 1.4–2.0 is feasible for initial predictions; a value over 2.0 is excellent for quantitative prediction. These evaluation parameters are calculated as follows:

(19) $$R_{c},R_{v},R_{p}=\sqrt{1-\frac{\sum_{i=1}^{n}(y_{p}-y_{i}{)}^{2}}{\sum_{i=1}^{n}(y_{i}-y_{p}{)}^{2}}}$$

(20) $$RMSEC,RMSEV=\sqrt{\frac{\sum_{i=1}^{n}\left(y_{p}-y_{i}\right)}{n}}$$

(21) $$MAE=\frac{\sum_{i=1}^{n}\left|y_{p}-y_{i}\right|}{n}$$

(22) $$Loss=Mse=\frac{\sum_{i=1}^{n}(y_{p}-y_{i}{)}^{2}}{n}$$

(23) $$RPD=\frac{1}{\sqrt{1-R_{p}^{2}}}$$

where *n* is the number of samples in the corresponding dataset (calibration dataset, validation dataset, and prediction dataset), and *y_p_*, and *y_i_* are the predicted rheological property values (extensibility and toughness) and reference rheological property values of the ith dough sample.

## 3. Results and Discussion

### 3.1. Statistics of Reference Extensibility and Toughness

**Figure 5:**
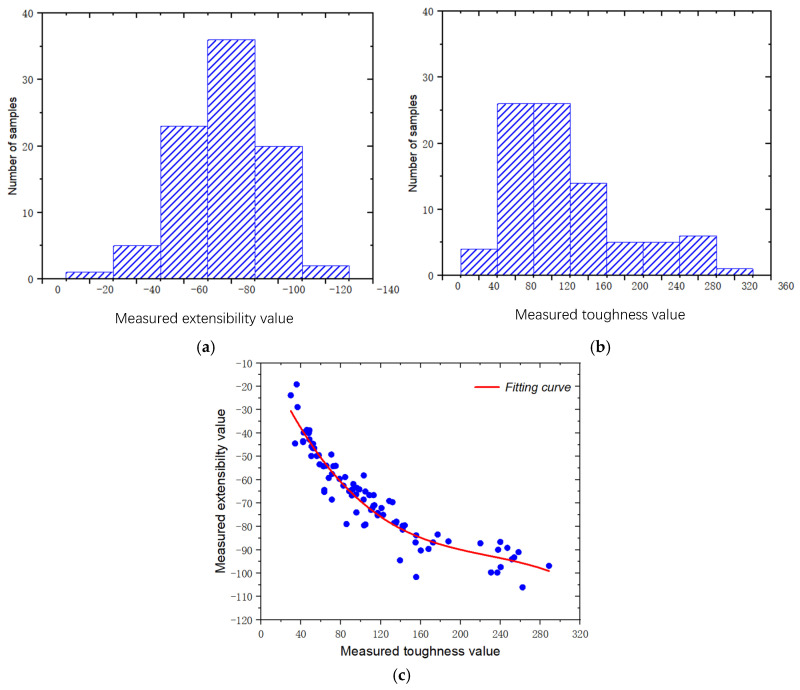
Statistical analysis of dough extensibility and toughness data. (**a**,**b**) are the distribution of standard measurement values for dough toughness and extensibility; (**c**) is the relationship between dough toughness and extensibility.

The distribution of 87 samples based on the mean values of standard measurement values for the toughness and extensibility of dough is shown in Figure 5. The measured values of extensibility and toughness of dough samples mainly follow a normal distribution. Their values of extensibility are mainly between −40 mm and −100 mm (Figure 5a). The range of their toughness values is primarily between 40 g and 160 g (Figure 5b). Additionally, Figure 5c depicts the relationship between measured extensibility and toughness. The figure demonstrates that the toughness of dough is positively correlated with the absolute value of dough extensibility. The greater the dough toughness, the greater the absolute value of dough extensibility. This was because the greater the toughness of the dough, the more gluten it contained, and the stronger its corresponding extensibility [1].

After the collected point cloud images were preprocessed, the collected images were then divided into training sample sets. The number of images in the calibration, validation, and test sets was 204, 51, and 51, respectively. The range, mean, and standard deviation (SD) of extensibility and toughness of the samples in calibration, validation, and prediction sets are listed in Table 1. The extensibility and toughness of all tested samples were normally distributed around the mean values of −71.913 mm and 120.456 g, respectively. In addition, the standard deviation values of the two indicators’ prediction sets were 18.463 and 64.448, respectively. Compared with the standard deviation of dough extensibility, the SD value of the prediction set of dough toughness fluctuated greatly. 

### 3.2. Training Analysis

**Figure 6:**
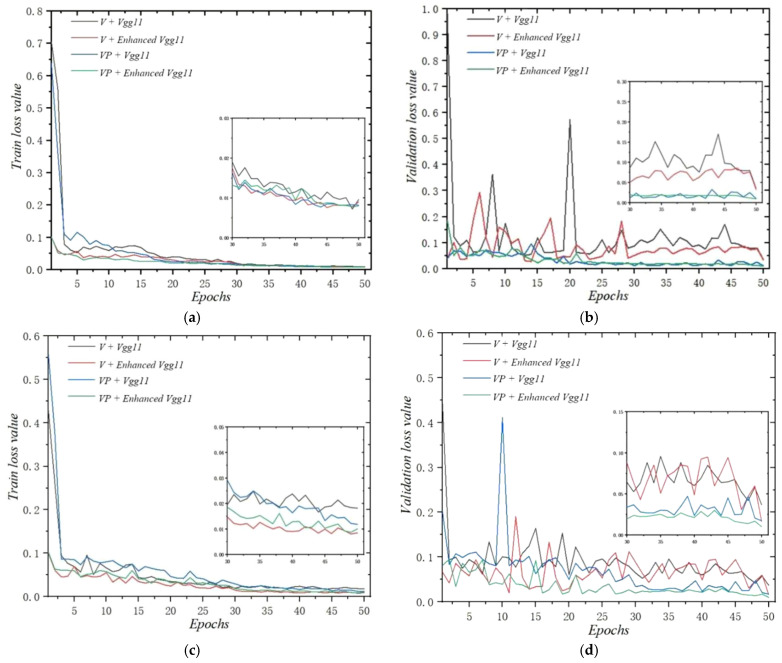
Training loss values of the different models and methods. (**a**,**b**) represent the loss values of training and validation for predicting dough toughness. (**c**,**d**) represent the loss values of training and validation for predicting dough extensibility. Note: V represents voxel-based data; VP represents voxel-point-based data.

Training losses and validation losses of the Vgg11 and enhanced Vgg11 using two different data-processing methods are plotted in Figure 6. According to the curve changes in Figure 6, it could be observed that the loss-value curves of the two models (Vgg11 and enhanced Vgg11) both decrease slowly with the increase in epochs until they converge and remain stable. The training loss value of the Vgg11 using voxel-based data (V) and voxel-point-based data (VP) decreases to below 0.09 after only five epochs of training. This training result indicated that Vgg11 has a good convergence effect. In addition, in the early stages of training, obviously, the loss values of V + enhanced Vgg11 and VP + enhanced Vgg11 were much lower than those of V + Vgg11 and VP + Vgg11, respectively. Furthermore, both V + enhanced Vgg11 and VP + enhanced Vgg11 are superior to V + Vgg11 and VP + Vgg11, respectively. Especially for voxel-based data, adding CBAM to the Vgg11 model could better improve its performance.

By comparing different loss-value curves of different models using voxel-point-based data or voxel-based data, it could be observed that both training models show a similar trend, and the training loss value of voxel-point-based data was not significantly better than that of voxel-based data during the training process, but the data validation loss value after voxel downsampling was much smoother and lower than that without sampling. The reason for this phenomenon was that voxel downsampling reduced the number of points and some unnecessary noise while maintaining the shape features and spatial structure information of the point cloud. Furthermore, the 3D convolution using voxels as point data as input was more conducive to the model’s ability to extract key features [37]. This training effect means that the models of VP + Vgg11 and VP + enhanced Vgg11 have more stable testing performance than Vgg11 and enhanced Vgg11, respectively.

### 3.3. Performance Comparison of Different Models

The training and validation results of different 3D CNN models are shown in Table 2. As shown in the table, the best training model among the four models (MobileNet [37], ResNet18 [38], Vgg11 [33], and enhanced Vgg11) is enhanced Vgg11. Although the *R_c_* of the four models was not significantly different, and even MobileNet and ResNet18 had slightly higher accuracy than Vgg11 and enhanced Vgg11 in predicting dough toughness with voxel-point-based data, and the accuracy of the validation set was much lower than that of Vgg11 and enhanced Vgg11. In addition, comparing the MobileNet and ResNet18 models, the ResNet18 model had a higher *R_v_* value than MobileNet. Moreover, for predicting the same dough indicators of rheological properties and using the same data-processing method, the training and validation times of the Vgg11, enhanced Vgg11, and MobileNet models were similar, but the training and validation time of ResNet18 for each epoch was much higher than the other models. Perhaps it was because the ResNet18 model needed to train far more parameters than the other two models. Furthermore, the CBAM was introduced to the Vgg11 model to improve the training performance, but it did not increase the training and validation time of the model.

### 3.4. Comparison Between Two Different Data-Processing Methods

The calibration and validation results of four different models using two different point cloud data-processing methods are shown in Table 2. Apart from the less obvious advantages of the MobileNet and ResNet18 models in predicting dough extensibility, processing voxels as points could significantly improve the calibration and validation results of the models. Especially for establishing Vgg11 and enhanced Vgg11 models using the voxels as point data, the *R_v_* of the models increased by over 0.082, and the *RMSV* of the models was reduced by over 0.073. This might be because when the spatial feature information was the same, complex data actually reduced the predictive performance of deeper models. Furthermore, inputting voxel-point-based data into the 3D CNN models improved the accuracy while also slightly reducing the training and validation times of the models, possibly because the data size had been reduced from 156 KB to 12 KB, which affected the training and validation time of each epoch of the model.

### 3.5. Analysis of Test Results

To further compare the testing performance of the trained enhanced Vgg11 with the other models using different data-processing methods, the additional 51 data samples were input into MobileNet, ResNet18, Vgg11, and Enhance Vgg11, respectively, and the test results are shown in Table 3. Except for the MobileNet model, all the other models had significantly improved their testing performance in predicting dough toughness by using voxel-point-based data. And the *R_P_* values of the ResNet18, Vgg11, and enhanced Vgg11 models increased by 0.145, 0.086, and 0.135, respectively. In terms of dough extensibility prediction, the models of ResNet18, MobileNet, Vgg11, and enhanced Vgg11 RP values rose by 0.108, 0.172, 0.122, and 0.127, respectively. This test result might be due to the fact that the performance of models trained using voxel-point data was more robust than that using voxel-based data. Under the same data-processing method, the test results of the Vgg11 and enhanced Vgg11 models were far superior to the other two models. The *R_P_* of the voxels as point data reached higher than 0.878, and the *RPD* reached over 2.090, indicating that the predictive performance of the models was good. The reason for this might be that the Vgg11 model has deeper and wider layers, smaller convolution kernels, *BN* layers, and two dropout layers added to its fully connected layer, which was beneficial for improving the prediction accuracy of the model, accelerating the convergence speed of the network, and preventing overfitting of the model [29]. Additionally, the testing performance of the enhanced Vgg11 model was also better than that of Vgg11. Because 3D CBAM can adaptively calculate the weights of different input data based on different parts and features of spatial deformation information, it makes the use of input data information more effective and improves model performance and robustness. Furthermore, compared to MobileNet, ResNet18 was overall slightly better than ResNet18. Perhaps it was because the network structure of ResNet18 was more complex than that of MobileNet.

**Figure 7:**
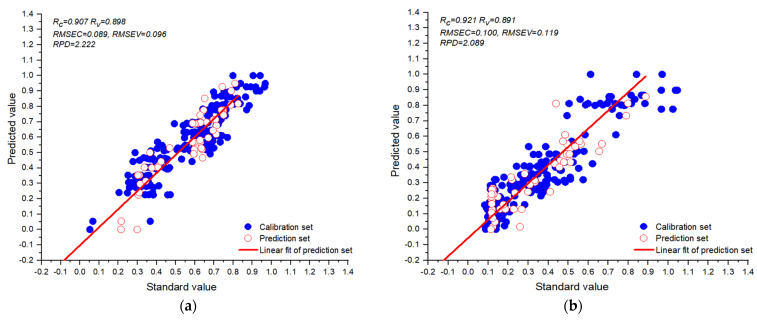
Enhanced vgg11 for predicting dough extensibility (**a**) and toughness (**b**).

Recently, some scholars have evaluated the strength-related properties and extensibility parameters of dough using a texture analyzer [39,40], mixograph analysis [4], brabender extensograph, and alveogram [6,36,37,38,39,40]. These instruments measure the rheological properties of dough from the perspective of mechanics. However, the deformation of dough during stretching is closely related to its rheological properties [12]. Another paper [1] first successfully applied the spatial deformation of sheeted dough using airflow-structured light 3D imaging technology to detect the rheological properties of dough. Furthermore, it also proved that the degree of spatial deformation of the dough bubble is related to the rheological properties of the dough [1,10,11]. Compared with this previous paper [1], our study did not need to go through complex and cumbersome point cloud data preprocessing, spatial deformation quantification, and prediction model construction. Furthermore, the prediction accuracy of the enhanced Vgg11 model for dough toughness and extensibility reached 0.878 and 0.893, respectively. And the RPD of the enhanced Vgg11 model for dough toughness and extensibility was 2.222 (Figure 7a) and 2.089 (Figure 7b), respectively. According to the performance evaluation criteria, RPD in the range of 2.0–2.5 indicates a good quantitative model [41]. However, the variety of flour and the number of dough samples used in this study are limited. In the future, the variety of experimental flour and the number of dough test samples can be expanded to establish a more adaptable and robust prediction model.

## 4. Conclusions

This study was conducted to evaluate dough extensibility and toughness using enhanced Vgg11 via bubble inflation and a 3D scanning imaging technique. The voxel-point-based data and voxel-based data were input into four different 3D CNN models, respectively. The prediction ability of the voxel-point-based data input model was better than that of the voxel-based data. In addition, by comparing the predictive performance of four different 3D CNN models, the enhanced Vgg11 was superior to the other three models. For dough extensibility, the R_c_, R_p_, and RPD of the enhanced Vgg11 model based on voxels as point data were 0.907, 0.893, and 2.22, respectively. Furthermore, the enhanced Vgg11 model for this study to predict dough toughness was based on voxel-point-based data and had an R_c_, R_p_, and RPD of 0.921, 0.878, and 2.089, respectively. These results indicated that introducing the CBAM into Vgg11 could improve the prediction performance of the Vgg11 model. In addition, the voxel-point-based data were input into enhanced Vgg11 models to evaluate the rheological properties of dough based on airflow, and a structured light 3D imaging technique achieved good prediction results. In the future, it is necessary to expand the variety of experimental flour and increase the number of dough test samples to establish a more adaptable and robust prediction model.

## Figures and Tables

**Table 1:** Statistics on toughness and extensibility of dough.

Indicators	Datasets	Number of Samples	Minimum	Maximum	Mean Value	Standard Deviation
Extensibility (cm)	Calibration set	204	−106.065	−19.140	−67.078	18.588
	Validation set	51	−106.065	−19.140	−65.916	18.774
	Test set	51	−101.625	−23.815	−71.913	18.463
Toughness (g)	Calibration set	204	29.9	288.725	115.711	67.003
	Validation set	51	32.17	251.51	118.868	60.278
	Test set	51	34.17	288.725	120.456	64.448

**Table 2:** The calibration and validation results of different models and methods.

Indicators	Method	Models	Calibration Set		Validation Set		Training Time/s (*Each Epoch*)	Evaluating Time/s (*Each Epoch*)	Occupies Space (*Each Data Point*)
			*R_c_*	*RMSEC*	*R_v_*	*RMSEV*			
Extensibility	V	MobileNet	0.941	0.086	0.449	0.208	87.22	17.34	156 KB
		ResNet18	0.916	0.085	0.588	0.187	664.51	43.02	
		Vgg11	0.921	0.098	0.591	0.188	86.38	16.15	
		E-Vgg11	0.927	0.095	0.629	0.181	84.76	15.99	
	VP	MobileNet	0.883	0.100	0.464	0.191	86.92	15.00	12 KB
		ResNet18	0.903	0.092	0.523	0.184	635.00	39.70	
		Vgg11	0.906	0.091	0.882	0.102	82.16	6.48	
		E-Vgg11	0.907	0.089	0.898	0.096	82.23	15.03	
Toughness	V	MobileNet	0.793	0.154	0.435	0.264	92.19	17.12	156 KB
		ResNet18	0.710	0.178	0.650	0.243	690.41	46.45	
		Vgg11	0.835	0.135	0.681	0.192	92.79	16.05	
		E-Vgg11	0.933	0.092	0.802	0.158	77.30	14.87	
	VP	MobileNet	0.935	0.091	0.485	0.249	80.49	16.49	12 KB
		ResNet18	0.946	0.083	0.845	0.152	666.07	43.02	
		Vgg11	0.904	0.109	0.886	0.132	73.42	14.82	
		E-Vgg11	0.921	0.100	0.891	0.119	56.79	14.47	

Note: V represents voxel-based data; VP represents voxel-point-based data; E-Vgg11 represents enhanced Vgg11.

**Table 3:** Test results of different 3D CNN models and methods.

Indicators	Methods	Models	*R_p_*	*MAE*	*RPD*
Extensibility	V	MobileNet	0.760	0.193	1.539
		Resnet18	0.685	0.155	1.373
		Vgg11	0.766	0.140	1.556
		E-Vgg11	0.766	0.138	1.556
	VP	MobileNet	0.868	0.147	2.014
		Resnet18	0.857	0.159	1.941
		Vgg11	0.888	0.127	2.175
		E-Vgg11	0.893	0.117	2.222
Toughness	V	MobileNet	0.735	0.189	1.475
		Resnet18	0.602	0.210	1.252
		Vgg11	0.699	0.162	1.398
		E-Vgg11	0.743	0.180	1.494
	VP	MobileNet	0.735	0.197	1.475
		Resnet18	0.747	0.131	1.504
		Vgg11	0.785	0.171	1.614
		E-Vgg11	0.878	0.128	2.089

Note: V represents voxel-based data; VP represents voxel-point-based data; E-Vgg11 represents enhanced Vgg11.

## Data Availability

The original contributions presented in the study are included in the article, further inquiries can be directed to the corresponding author.
